# Capturing the variety of clinical pathways in patients with schizophrenic disorders through state sequences analysis

**DOI:** 10.1186/s12874-023-01993-7

**Published:** 2023-07-29

**Authors:** Laura Savaré, Francesca Ieva, Giovanni Corrao, Antonio Lora

**Affiliations:** 1 MOX - Department of Mathematics, Politecnico di Milano, Milan, Italy; 2 HDS, Health Data Science Center, Human Technopole, Milan, Italy; 3 National Centre for Healthcare Research and Pharmacoepidemiology, University of Milano-Bicocca, Milan, Italy; 4 Unit of Biostatistics, Epidemiology and Public Health, Department of Statistics and Quantitative Methods, University of Milano-Bicocca, Milan, Italy; 5 Department of Mental Health and Addiction Services, ASST Lecco, Lecco, Italy

**Keywords:** State sequence analysis, Care pathways, Schizophrenic disorder

## Abstract

**Background:**

Care pathways are increasingly being used to enhance the quality of care and optimize the use of resources for health care. Nevertheless, recommendations regarding the sequence of care are mostly based on consensus-based decisions as there is a lack of evidence on effective treatment sequences. In a real-world setting, classical statistical tools were insufficient to consider a phenomenon with such high variability adequately and have to be integrated with novel data mining techniques suitable for identifying patterns in complex data structures. Data-driven techniques can potentially support empirically identifying effective care sequences by extracting them from data collected routinely. The purpose of this study is to perform a state sequence analysis (SSA) to identify different patterns of treatment and to asses whether sequence analysis may be a useful tool for profiling patients according to the treatment pattern.

**Methods:**

The clinical application that motivated the study of this method concerns the mental health field. In fact, the care pathways of patients affected by severe mental disorders often do not correspond to the standards required by the guidelines in this field. In particular, we analyzed patients with schizophrenic disorders (i.e., schizophrenia, schizotypal or delusional disorders) using administrative data from 2015 to 2018 from Lombardy Region. This methodology considers the patient’s therapeutic path as a conceptual unit, composed of a succession of different states, and we show how SSA can be used to describe longitudinal patient status.

**Results:**

We define the states to be the weekly coverage of different treatments (psychiatric visits, psychosocial interventions, and anti-psychotic drugs), and we use the longest common subsequences (dis)similarity measure to compare and cluster the sequences. We obtained three different clusters with very different patterns of treatments.

**Conclusions:**

This kind of information, such as common patterns of care that allowed us to risk profile patients, can provide health policymakers an opportunity to plan optimum and individualized patient care by allocating appropriate resources, analyzing trends in the health status of a population, and finding the risk factors that can be leveraged to prevent the decline of mental health status at the population level.

## Background

Diagnostic-therapeutic pathways are evidence-based interventions aimed to organize the healthcare assistance process for specific groups of patients and to enhance the quality of care across the continuum by improving risk-adjusted patients outcomes, promoting patient safety, increasing patient satisfaction, and optimizing the use of resources [1]. Recommendations regarding the sequence of care are mostly based on consensus-based decisions as there is a lack of evidence on effective treatment sequences and consequent high variability in treatments. In chronic diseases, studying the effect of these patterns on adverse outcomes is of clinical relevance. Nevertheless, the way they are accounted for in predictive models is far from being as informative as it may be [2–4]. In the field of epidemiological research, the use of administrative health databases has now become a widely used strategy thanks to the always greater reliability of the detection methodologies adopted, which involve the acquisition of high-quality data [5]. These databases also offer an ever-increasing amount of useful information that allows to conduct epidemiological studies with less resources and cost and time savings. One of the main features of the studies conducted with such databases concerns the possibility of reflecting real clinical practice on large and unselected populations, an aspect that surely exceeds one of the main limits of randomized clinical trials. Although they offer a growing amount of useful information, to date, studies focusing on individual pathways have mainly remained descriptive, without taking into account the possible evolution of care consumption over time [6, 7]. Although in recent years, some methodologies were proposed to tackle this kind of information (such as the trajectories models [8], temporal association rules [9], supervised machine learning [10], the tree-based scan statistic [11], and the latent class model [12–14]), they are not always suitable to evaluate complex longitudinal patterns of care and to be implemented on real-world data. For example, they usually work on a limited number of class-defining variables and are thus unsuitable for evaluating complex longitudinal patterns of prescriptions. Furthermore, pragmatic aspects, such as the need for stakeholders to understand the method in order to be confident in the results, must be considered. In this framework, classical statistical tools must be integrated with novel data mining techniques to identify patterns in complex data structures properly. Data-driven techniques can potentially support empirically identifying effective care sequences by extracting them from data collected routinely in health care [15].

State Sequence Analysis (SSA) is an upcoming technique in epidemiology, derived from social sciences, that could provide useful insights on the chronology of care consumption, the interval and timing of treatment patterns used in the clinical practice and the effectiveness of different treatment sequences. SSA also allows for the identification of specific patterns in the delivery of healthcare assistance [16]. Specifically, attention is focused on the ordered sequence of states (or activities) experienced by individuals over a given time span (usually at T equally spaced discrete time periods). To this end, pairwise dissimilarities among sequences in their entirety are first assessed. Dissimilarity matrices are then employed to identify the most typical trajectories using cluster analysis. Applied to data recorded in health care information systems, a field of application which is still almost unexplored, this technique can assess performed medical behavior and the chronological sequence of these behaviors, allowing to identify the most relevant patterns in the data and assess the most efficient one in preventing adverse events.

In this work, a novel pipeline for using SSA in the health area in order to monitor and evaluate the diagnostic-therapeutic paths of patients is proposed and discussed. It is then applied to the pathway of patients affected by *schizophrenic disorder*. Throughout the text, with “schizophrenic disorder” or “schizophrenia spectrum disorder”, we mean those taken in charge by the facilities of the Department of Mental Health with a diagnosis of “schizophrenia, schizotypal or delusional disorders”, as other authors did [17, 18]. The choice of this application is motivated by the fact that, in mental health, problems are frequently encountered in the quality of care provided [19, 20]. The care pathways of patients with severe mental disorders often do not correspond to the standards required by the evidence in this field, and they present a high variability between countries and within them. Moreover, the lack of definition of optimal diagnostic-therapeutic paths has prompted us to investigate two key aspects. Firstly, we aim to determine if a common pattern of care can be identified, enabling health policymakers to plan optimal and individualized patient care by allocating appropriate resources. Secondly, we seek to analyze trends in the population’s health status and identify modifiable risk factors that can help prevent a decline in overall health at the population level. In a real-world setting, state-of-the-art tools resulted in insufficiently considering a phenomenon with such high variability [17, 21], and it is also difficult to find the statistical methodology suitable to consider so many different items properly. For these reasons, it is very important to outline synthetic tools in order to provide process indicators.

In particular, there is the need to describe and analyze longitudinal care pathways to then evaluate their association with the incidence of negative outcomes. Indeed, only in recent years European countries increasingly recognized how important it is to equip policymakers, care providers, and service users in a health system with tools to move towards a person-centered system, and improvement is being made on what it actually means to deliver person-centered mental health care. This became a priority also at the European level, as testified by a number of projects and initiatives aimed at pursuing these goals, such as the JA ImpleMENTAL, a European Joint Action born with the purpose of providing support for member states’ implementation of best practices in the area of mental health. In fact, in Europe, only a few countries currently use indicators to routinely assess the quality of mental health care, and real-world data collected in Healthcare utilization (HCU) databases may represent essential leverage for the quality improvement of mental health care. Right now, there are very few attempts to build these indicators to monitor services, and an evaluation process is not yet used in the routine practice [18]. Therefore, clinical indicators are urgently needed since they are a useful tool to address the purpose of monitoring and evaluating the quality of care provided by the National Health Service (NHS), specifically in the field of mental health [22, 23]. The aim of the present work is to provide a reference method to allow the governance to monitor over time, assess the quality and optimize the diagnostic-therapeutic pathways. This knowledge could be used for (re)designing and optimizing existent care pathways.

The paper is structured as follows. Methods section introduces the SSA methodology and its steps, a description of the dataset, data extraction, inclusion criteria, and the data setting. In particular, the data coding and the pathway construction, the representation of the sequences, the description of the unidimensional indicators summarizing longitudinal characteristics of the sequences, the dissimilarity measures to compare sequences and the sequences profiling via unsupervised procedures, and finally, the posthoc analysis and prediction models using sequences’ clusters and unidimensional indicators are addressed. Key results from applying these methods to administrative data for patients affected by the schizophrenic disorder are presented in the Results section. We then discuss the strengths of representing diagnostic-therapeutic pathways as sequences through administrative data in a state sequence analysis framework and the limitations of the current approach, which open doors to further developments in this area. Finally, we end with the main conclusions and explain the importance and relevance of the study reported.

Statistical analyses were performed in the R-software environment [24], using TraMineR package for mining and visualizing sequences of categorical data [25].

## Methods

This work is concerned with categorical sequence data and, in particular, with state sequences in which the position of each successive state is given a meaningful interpretation in terms of age, date, or more generally of elapsed time or distance from the beginning of the sequence. In SSA terminology, a care path corresponds to a sequence in which successive “states,” i.e., the value taken by the variable at a time t, are encountered. The term “sequence” is therefore used to refer to the entire care pathway and, applied to data collected in health care information systems, can assess the diagnostic-therapeutic pathway of patients. Thus, the main goal of sequence methods is to extract simplified actionable information from sequential data sets, i.e., to efficiently summarize and represent these sets and categorize the sequential patterns into a limited number of groups. This is essentially an exploratory task, consisting of computing summary indicators and sorting, grouping, and comparing sequences. The resulting groups and real value indicators can then be subjected to classical inference methods and serve, for example, as response variables or explanatory factors for regression-like models.

### Object oriented representation: identifying of states and sequences

**Fig. 1:**
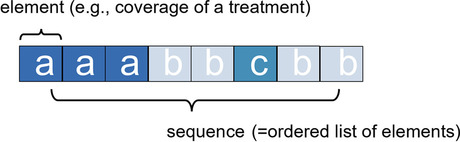
Example of sequence

State sequences can be represented in many different ways, depending on the data source and how the information is organized [26]. To perform SSA, it is usually necessary to first convert the data to a state sequence format, where each row represents a conceptual unit (e.g., a patient). Data organization and conversion between formats are discussed in detail in [27], where an ontology for representing longitudinal data is also given. Formally, a state sequence of length *l* is defined as an ordered list of *l* elements selected sequentially from a finite set A of size $a = \left| {A}\right|$, namely *alphabet*, containing all possible states occurring in the data. An element can be a particular state (e.g., employment, health status, or coverage of a treatment ), a physical object (e.g., base pair of DNA, protein, or enzyme), or an event (e.g., hospitalization). The positions of the elements are fixed, ordered by elapsed time, and they refer to a relative, not absolute, point in time. A natural way of representing a sequence x is to list the successive elements that make up the sequence $x = (x_1, x_2, . . . , x_\textit{l})$, with $x_j \in A$ (Fig. 1). The methods addressed refer to sets of sequences, each considered a whole, i.e., a conceptual unit.

Once the alphabet has been established, the length of the study period and the time unit (e.g., day, week, month, etc.) of the analysis must be determined, also referred to as the “sequence granularity.” These two aspects determine the beginning, the end, and thus the length of the sequences to be analyzed. The complexity of the analysis is increased by adding sequential information in the research design due to the exponential growth in the number of possible sequences with increasing sequence length. Thus, dealing with sequence data raises two questions (i) how to maintain the sequential nature of the data without reducing it to single events, (ii) and how to optimally reduce the variation in the sequences. SSA can answer these questions by visualizing the sequences, computing some indicators of the features of each sequence, comparing different sequences using distance measures, and grouping the sequences.

### Defining process indicators via suitable sequence representation

Sequences can be visualized by a sequence index diagram. It represents them by horizontally stacked boxes colored according to the state at successive positions. In this way, the individual sequence of states in the longitudinal direction becomes visible for each case, as well as - by the length of each colored segment - the time spent in each state. The arrangement also allows simple cross-comparisons at each position. However, with a large number of sequences, no specific patterns can be identified, and interpretation is also difficult. Other options for visualizing sequences include frequency and representative plots [25]. In sequence frequency plots, sequences are sorted by their frequency in the dataset, and usually, the ten most frequent sequences are shown with a bar width proportional to the frequency, while in representative sequence plots, sequences are sorted by their representativeness value. Finally, the state distribution plot shows the proportion of states at each time point. An interesting summary that can be derived from the state distributions is the sequence consisting of the most frequent state at each position (i.e., modal states) [25].

### Sequences’ longitudinal characteristics

Other important characteristics of sequences can be described by the number of states in each sequence, the mean time spent in each state, and the number of transitions between states. In addition, some indicators are also defined to summarize the longitudinal features of individual sequences, such as entropy, turbulence, and sequence complexity [27]. In particular, in the context of information theory and statistics, entropy refers to the measure of uncertainty or randomness in a set of data or a system. It quantifies the average amount of information or surprise contained in each event or data point. , The concept of turbulence, instead, depends on the number of transitions and/or the number of distinct states and/or the variation in the timing/duration of events. Sequence complexity refers to the degree of intricacy or diversity within a sequence of data elements. It measures the richness or variety of patterns, structures, or information content present in a given sequence.

In this work, we used the longitudinal Shannon entropy, which can be considered a measure of uncertainty in predicting the next state within a data sequence:

1 $$\begin{aligned} h(\pi _1,...,\pi _\alpha )= -\sum _{i=1}^{a}\pi _i log(\pi _i) \end{aligned}$$

where *a* is the size of the alphabet and $\pi _i$ is the fraction of occurrences of the *i*-th state in the sequence under consideration. Therefore, entropy is 0 when all states of a sequence are equal; it is maximal when a sequence consists of all states in equal proportions. Elzinga & Liefbroer’s first measured sequence turbulence (2007). It is based on the quantity $\phi (x)$ of distinct subsequences that can be derived from the succession of various consecutive states as well as the variance of the repeated intervals $t_i$ between the various states. For a sequence x, the formula is

2 $$\begin{aligned} T(x) = log_2 \bigg {(}\phi (x)\frac{s^2_{t,max}(x)+1}{s^2_t(x)+1}\bigg {)} \end{aligned}$$

where $s_t^2(x)$ is the variance between successive state durations in the sequence *x* and $s_{t,max}^2(x)$ is the maximum value this variance can take given the number of states and the total duration of the sequence. From a prediction point of view, the larger the differences in the state durations, and hence their variance, the more uncertain the sequence. In this sense, a small variance in duration implies high complexity.

### Measuring dissimilarity and clustering sequences

Dissimilarity measures between sequences can be divided into measures based on the (minimal) cost of converting one sequence to the other and those defined as the number of matching attributes. Another interesting distinction is between measures that perform positional comparisons, i.e., that do not allow a sequence or part of it to be shifted, and those that consider similar shifted patterns [28]. The optimal matching algorithm (OM) is the most widely used technique [29] in the social sciences. OM is a family of dissimilarity measures originally proposed by Levenshtein (1965) from the field of information theory and computer science and adapted [30] to the analysis of CVs by Abbott (1995). Basically, OM expresses the distances between sequences in terms of the minimum effort required to transform one sequence into the other, as measured by the subsequent operations. Within this framework, three basic sequence conversion operations are possible: insertion (a state is added into the sequence), deletion (a state is removed from the sequence), and substitution (another state replaces a state). Specific costs can be assigned to each of these elementary operations. Typically, the insertion and deletion cost is set to 1, while the replacement cost can vary. In this way, the distance between two sequences can be defined as the minimum cost of converting one sequence to another. However, defining this cost often involves subjective choices that can lead to triangular inequality violations if not properly tuned. Several proposals in the literature have introduced criteria to improve or guide the choice of costs in OM. For example, data-driven estimation of the substitution cost matrix using the transition rates between states, i.e., using substitution costs inversely proportional to the transition frequencies between two states [31]. Consider two states *a* and *b*. Let $N_t(a)$ and $N_t(b)$ be the number of individuals experiencing respectively *a* and *b* at time *t*, and $N_{t,t+1}(a,b)$ be the number of individuals experiencing *a* at time *t* and *b* at time $t+1$. The transition frequency from *a* to *b* is

3 $$\begin{aligned} p_{t, t+1}(a,b) = \frac{\sum _{t=1}^{T-1}N_{t, t+1}(a,b)}{\sum _{t=1}^{T-1}N_{t}(a)} \end{aligned}$$

The cost of replacing *a* with *b*, *c*(*a*, *b*), can be defined as $c(a,b) = c(b,a) = 2-p_{t, t+1}(a,b)-p_{t,t+1}(b,a)$. This cost specification takes into account the occurrence of events and gives more weight to the less frequent transitions. Due to the fact that transitions are qualitatively different at different times, Lesnard (2006) proposed a dynamic Hamming distance based on time-varying substitution costs $c_t(a,b)$ [29]. Moreover, these editing operations have no direct interpretation in environments other than bioinformatics, making it difficult to obtain meaningful results. In particular, insertion and deletion operations distort time to find identically encoded states but occur at different times in the respective sequences. A simple solution to avoid insertion and deletion operations is to use the Hamming distance, which measures the minimum number of substitutions required while preserving the original time scale [32]. While focusing on substitution operations has the sociological advantage of targeting trajectories with simultaneous similarities, unlike the forbidden insertion and deletion operations that focus on matching states regardless of their timing, this distance may suffer from temporal rigidity because anticipations and/or shifts of the same decisions in the life course are not taken into account. Therefore, similar sequences that are shifted over a period of time may be maximally dissimilar, whereas the dissimilarity is less problematic for sequences that remain in the same state for a long time. Alternative dissimilarity criteria have also been introduced to control for the importance assigned to the characteristics of sequences (i.e., the collection of states experienced, their timing, or their duration) when assessing their differences [33].

**Fig. 2:**
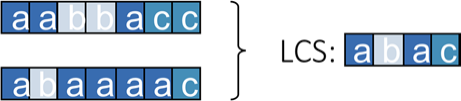
The Longest Common Subsequence between two sequences

Later, the longest common subsequence (LCS) metric was introduced as a special case of OM [28]. According to Elzinga’s proposal, two sequences are very similar if they have long subsequences in common, with gaps allowed between elements (Fig. 2). In this way, stable patterns in the sequences can be extracted, and the length of the shared subsequences can be used as an indicator of the similarity of two strings. The main difference is that OM distances identify the part of a sequence that needs to be changed to match another sequence, while LCS identifies the parts that are already sequentially common for both sequences. Therefore, the result of applying OM between two sequences is a number that measures how similar two patterns are, and the result of applying LCS is a subset of the states that belong to the common subsequence that extracts stable patterns followed by individuals. However, if the next step is to cluster the sequences, we definitely need to create a distance matrix, and the basic OM (with substitution cost equal to 2) and LCS are the same. Let *A*(*x*, *y*) be a number of common attributes between sequences *x* and *y* that occur in the same order. It is an approximation measure because the higher the number, the closer the sequences are to each other. We derive a dissimilarity measure by the following general formula:

$$d(x, y) = A(x, x) + A(y, y) - 2A(x, y)$$

where *d*(*x*, *y*) is the distance between objects *x* and *y*. The dissimilarity is maximal when $A(x, y) = 0$, i.e., when the two sequences have no attribute in common. It is zero if the sequences are identical, i.e., if $A(x, y) = A(x, x) = A(y, y)$. In particular, note that a dissimilarity measure quantifies the distance between two sequences or, more generally, between two objects. This quantification makes it possible to compare the sequences or, for example, to set up a dissimilarity matrix for cluster analysis.

Compared to metrics based on position, the LCS metric reduces distances by accounting for non-aligned matches, i.e., position-shifted similarities. It should be noted that this methodology is purely sequential and does not consider temporal dimensions. Nevertheless, there are no results that prove that one method is superior to others, and the choice of dissimilarity measure remains a problem in SSE analysis. In what follows, we have adopted the LCS metric to account for similar patterns with similar subsequences, even when translated over time.

After computing a dissimilarity matrix ${\textbf {D}}$ obtained from a set of sequences $S = (s_1,..., s_n)$, where *n* is the number of subjects, cluster analysis can be applied to group sequences and identify the most typical trajectories traversed by the studied individuals. Heuristic clustering algorithms, either hierarchical or partial, are usually used. All cluster analyses produce results, regardless of their relevance. It is therefore necessary to discuss their quality in order to specify the scope of the results and not to make unwarranted generalisations. We performed a cluster analysis using a hierarchical clustering method, i.e., Ward’s algorithm [34]. Our goal was to make a posterior interpretation of the results of the cluster analysis in order to profile the groups of patterns and, if necessary, to calculate the association between the obtained grouping and other variables of interest.

### Post-hoc analysis

In many applications, the interest is to relate sequences to a set of covariates and/or endpoints of interest. In this framework, the resulting groups can then be subjected to classical inference methods and serve as response variables or explanatory factors for regression-like models. For example, one might want to know whether the trajectories of young individuals differ significantly from those of older individuals or whether gender has an effect on the trajectories. For this purpose, the relationship between sequences and stratifying factors can be tested with a chi-square test or logistic regression. Multiple covariates can be included, for example, by constructing a sequence regression tree, or multifactorial discrepancy methods can be used to test for differences between groups [35]. In this work, we performed a posthoc analysis to characterize the clusters obtained by a traditional hierarchical method, but any distance-based methods can also be used.

Furthermore, the results of sequence mining techniques can be integrated into classical statistical models to assess the association between specific patterns and health-related outcomes [36]. In this work, clusters identified by SSA during the first year after schizophrenia, schizotypal or delusional disorder diagnosis and groups based on complexity measures (high/low longitudinal entropy and turbulence) were used as independent variables to predict subsequent hospitalizations in a time-to-event analysis.

**Fig. 3:**
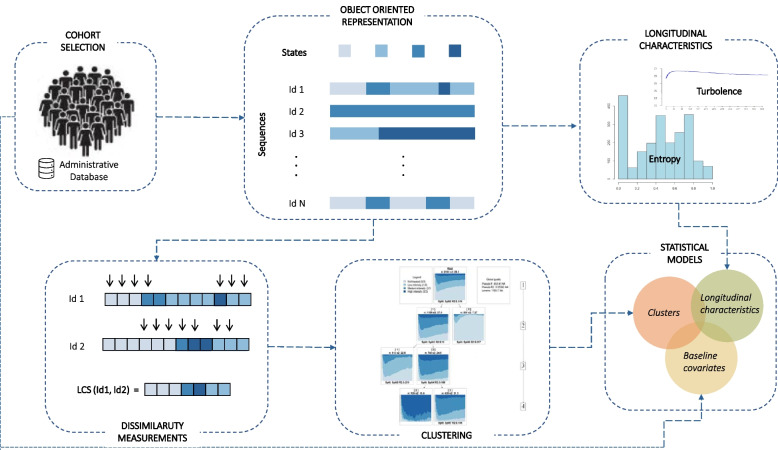
Algorithm pipeline of the methodology applied in this study: the SSA approach

A schematic graphical representation of the entire process flow is shown in Fig. 3.

### Data sources

Data used for this study were obtained from health care utilization databases in Lombardy, a region of Italy with approximately 16% (10 million) of the population. The Italian population is covered by the NHS, which provides hospitalization, major diagnostic procedures, and so-called life-saving drugs, including antipsychotics, free or almost free of charge to all citizens. In addition, a dedicated automated mental health care system collects data from regional Departments of Mental Health (DMHs) accredited by the NHS. This system provides demographic information and diagnosis codes for patients receiving specialized psychiatric care [37]. Specifically, public DMHs, which are arranged into a network of community services including Community Mental Health Centres, Day-Care Centers, General Hospital Psychiatric Wards, and Community Residential Facilities, provide the majority of the mental healthcare for patients with schizophrenic disorder. According to Corrao et al. [38], only a small percentage of patients with a diagnosis of schizophrenia spectrum disorder receive care from private facilities. Patients with schizophrenic disorders have free access to public DMHs. Twenty-six categories of interventions and activities provided by DMHs in outpatient, home care, or daycare facilities were coded and classified into two broad categories: “psychosocial interventions” and “generic care” [38]. Because a unique identification code is used for all databases within each region, the complete care pathway of NHS beneficiaries can be obtained through record linkage. Further details on HCU database used in the field of mental health have been reported in [17, 39].

### Study setting

The target population of this work consisted of all NHS beneficiaries resident in Lombardy, aged 18-40 years old, who during the recruitment period (January 2015 - December 2018) had at least one contact with a mental health service and had a diagnosis of schizophrenia, schizotypal or delusional disorders. We then excluded prevalent patients, namely those patients who already had such diagnosis before January 2015, and those with less than one year of follow-up. The patients included in the final cohort were followed for one year after the onset of the schizophrenic disorder (i.e., without experiencing death, emigration, or hospitalization during the first year after diagnosis). During the first year, we collected all psychiatric visits, psycho-social interventions (which consisted mostly of psychotherapy sessions), and anti-psychotic dispensed drugs dispensed to each patient. The combination of these three interventions makes up the optimal treatment pathway (OTP).

Diagnostic and therapeutic codes and service interventions and activities classified in the Italian Mental Health Information System used in the current study for drawing records and fields from HCU databases are reported in Tables 1 and 2, respectively. The codes are reported using the International Classification of Diseases (ICD-9-CM, ICD-10), and the Anatomical Therapeutic Chemical Classification System (ATC).

**Table 1:** Diagnostic and therapeutic (ICD-9-CM, ICD-10, and ATC) codes used in the current study for drawing records and fields from Healthcare Utilization databases

Schizophrenia	ICD-10 codes (Lombardy)
Schizophrenia	F20.*
Schizotypal disorder	F21.*
Delusional disorders	F22.*
Brief psychotic disorder	F23.*
Shared psychotic disorder	F24.*
Schizoaffective disorders	F25.*
Other psychotic disorder not due to a substance or known physiological condition	F28.*
Unspecified psychosis not due to a substance or known physiological condition	F29.*
Drugs	ATC codes
Antipsychotic agents	N05A (excluded N05AN)

**Table 2:** Service interventions and activities classified in the Italian Mental Health Information System

	Italian Mental Health Information system codes
Psychosocial interventions	
Individual living skills training	16
Group living skills training	17
Individual socialization	18
Group socialization	19
Single family psychoeducation	13
Multifamily group psychoeducation	14
Individual bodywork (i.e., expressive, practical manual and motor intervention), leisure activities	21
Group bodywork (i.e., expressive, practical manual and motor intervention), leisure activities	22
Work training	23
Assistance with financial and welfare procedures	25
Psychotherapy	
Psychological interview	02
Individual psychotherapy	07
Couple psychotherapy	08
Family psychotherapy	09
Group psychotherapy	10
Psychiatric visit	01

## Results

### Definition of the states and cohort selection

In our case study, we defined the states of the sequence for each patient on a weekly basis. Based on the OTP, we classified each state every week into four categories, based on how many of the three treatments (i.e., psychiatric visits, psycho-social interventions, and anti-psychotic) were dispensed to the patients:

- *Not treated (0 out of 3)*, if in that given week, the patient is not covered by any of the three treatments;
- *Low variety of treatments (1 out of 3)*, if covered by only one treatment;
- *Medium variety of treatments (2 out of 3)*, if covered by two treatments out of three;
- *High variety of treatments (3 out of 3)*, if covered by all three treatments.

In particular, we considered for each psychiatric visits a coverage of 2-4 weeks, depending on who provided the visit, for each psycho-social intervention a coverage of 2 weeks, and for the anti-psychotic drugs the defined daily dose.

Out of the 8’566 patients aged 18-40 years and assisted by the NHS for schizophrenic disorder in the years 2015-2018, 2’739 were incident users. Among these, only 2’329 patients had at least one year of observation and were included in the final cohort. The patients’ characteristics are reported in Table 3. In particular, we reported a few baseline characteristics including sex, age, and the number of co-treatment, dispensed during the two years before the date of the diagnosis of schizophrenia spectrum disorder, which was assessed and categorized as 0, 1 - 2, $\ge 3$. In addition, the clinical status of the patients was quantified by the Multisource Comorbidity Score (MCS), a prognostic score, based on hospitalization and dispensed drugs, that has been shown to be a good predictor of all-cause mortality and hospitalization of the Italian population [40]. The strata identified by the MCS are: *good* (score=0), *medium* (1$\le$score$\le$4), and *poor* (score$\ge$5). Finally, we collected all the considered treatments (i.e., psychiatric visits, psycho-social interventions, and anti-psychotics) during the first year of follow-up.

**Table 3:** Characteristics of the cohort members

	Cohort members
	(*N* = 2,329)
Male gender	1,449 (66.1%)
Age class (years at index date)	
18 - 29	1,204 (54.9%)
30 - 40	989 (45.1%)
Number of co-treatments:	
0	1,446 (65.9%)
1-2	719 (32.8%)
$\ge 3$	28 (1.3%)
Clinical status^a^	
Good	1,065 (48.6%)
Intermediate	192 (8.8%)
Poor	936 (42.7%)
Weekly variety of treatments ^b^: mean (SD)	
No treatments	24.1 (20.2)
Low	13.1 (12.6)
Medium	10.9 (12.9)
High	3.9 (9.6)

^a^Clinical status was assessed by the MCS, and 3 categories were considered: good (score=0), medium (1$\le$score$\le$4), poor (score$\ge 5$);

^b^Treatments are considered during the first year of follow-up

The majority of these patients were male (66.1%), more frequently under 30 years old. Also noticed a low number of co-treatments, also being patients under 30 years old, but 42.7% of them resulted in having a poor clinical status (i.e., with a low MCS). During the first year, on average, patients were not covered by any treatment for 24 weeks (approximately 6 months). For about a month, they were covered by 1 or 2 types of treatment, and only for a month did the variety of treatments become high, indicating that patients were fully covered with all the OTP’s treatments.

### Sequences’ characterization

The transition rates between states, are shown in Table 4. The most stable state is the *no treatment* one, (*0 out of 3*), with a probability of remaining in this state, for those who are already there, equal to 95%. On the other hand, for the other three states, the probability of remaining in the same state is 81%, 83%, and 87%, respectively. Furthermore, we see the highest transition rate for the patient that discontinued treatment, i.e., from *2 out of 3* to *1 out of 3* and from *3 out of 3* to *2 out of 3* state, that is equal to 12%. Finally, as expected, patients tend to add or discontinue treatment at once, tending the transition rate from *3 out of 3* to *0 out of 3* or *1 out of 3* state to zero.

**Table 4:** Transition Matrix

	-> (0/3)	-> (1/3)	-> (2/3)	-> (3/3)
(0/3) ->	0.95	0.04	0.01	0.00
(1/3) ->	0.09	0.81	0.10	0.01
(2/3) ->	0.01	0.12	0.83	0.04
(3/3) ->	0.00	0.01	0.12	0.87

**Fig. 4:**
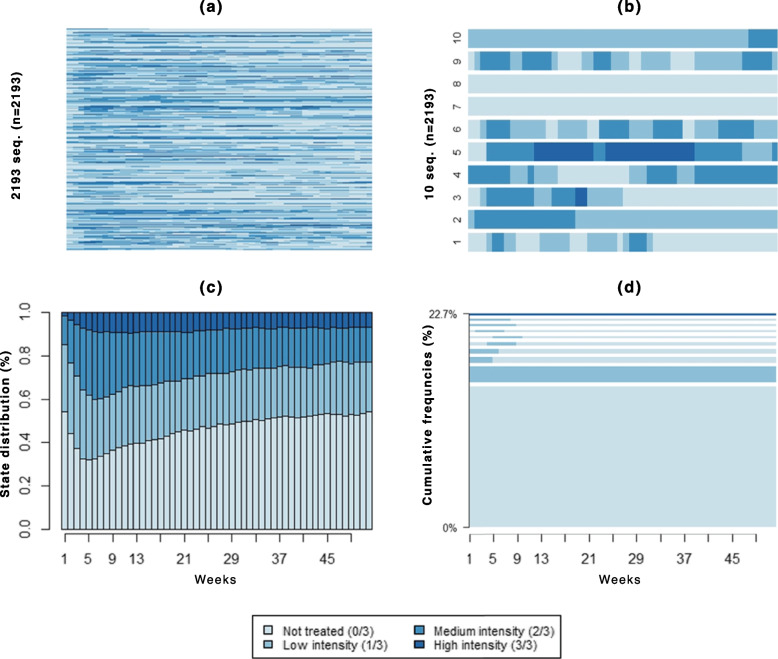
Different visualization of sequences. **a** Index plot; **b** Sequences of the first ten patients; **c** State distribution plot; **d** Frequency plot

In the top panel of Fig. 4, the sequences of each cohort member (a) and of the first 10 patients (b) are displayed. The high variability between patients and the inadequacy of most of the patterns can be noticed. The state distribution plot (c) shows a high proportion of patients without any treatments during all the weeks, being almost always equal to or greater than 50%. Only around the end of the first month the proportion of patients treated with *2 out 3* treatments seems to be increased, but soon many patients discontinue the treatments initiated. During the first year after the diagnosis of schizophrenia spectrum disorder, 18% of patients did not receive any treatment, and with the exception of the few patients who remain treated with at least one treatment throughout the year, plot (d) shows many sequences representing patients who started treatment around the first month of follow-up, but then have it soon discontinued.

### Profiling sequences

The calculation of the distance through the LCS metric and the study of the optimal number of groups to be considered allowed us to cluster our patients into three different groups.

Table 5 shows the distributions of variables across clusters, that can be used to perform an a-posteriori profiling of the clusters. Males and females are equally distributed among clusters, whereas the third cluster resulted to be more represented by young patients, but with the worst clinical conditions and with the highest percentage of co-treatments, despite their young age. Finally, we can characterize the 3 groups as:

- 1237 patients with *very low variety* of treatment [1];
- Six hundred sixty-two patients with *medium-low variety* of treatment [2];
- Two hundred ninety-four patients with *medium-high variety* of treatment [3].

**Table 5:** Characteristics of the cohort members by clusters

	Cluster 1	Cluster 2	Cluster 3	*P*-value
	(*N* = 1,237)	(*N* = 662)	(*N* = 294)	
Male gender	799 (64.6%)	443 (66.9%)	207 (70.4%)	0.143
Age class (years at index date)				
18 - 29	632 (51.1%)	351 (53.0%)	221 (75.2%)	< 0.001
30 - 40	605 (48.9%)	311 (47.0%)	73 (24.8%)	
Number of co-treatments:				
0	866 (70.0%)	407 (61.5%)	173 (58.8%)	<0.001
1-2	351 (28.4%)	247 (37.3%)	121 (41.2%)	
$\ge 3$	20 (1.6%)	8 (1.2%)	0 (0.0%)	
Clinical status^a^				
Good	636 (51.4%)	324 (48.9%)	105 (35.7%)	<0.001
Intermediate	121 (9.8%)	57 (8.6%)	14 (4.8%)	
Poor	480 (38.8%)	281 (42.5%)	175 (59.5%)	
Weekly variety of treatments ^b^: mean (SD)				
No treatments	39.6 (12.2)	5.0 (5.9)	1.9 (3.0)	<0.001
Low	9.0 (9.6)	23.0 (13.7)	7.5 (6.5)	
Medium	2.9 (5.0)	22.8 (13.9)	17.9 (8.8)	
High	0.5 (2.2)	1.2 (2.9)	24.7 (12.2)	

^a^Clinical status was assessed by the MCS, and 3 categories were considered: good (score=0), medium (score 1 - 4), poor (score$\ge 5$);

^b^Treatments are considered during the first year of follow-up

**Fig. 5:**
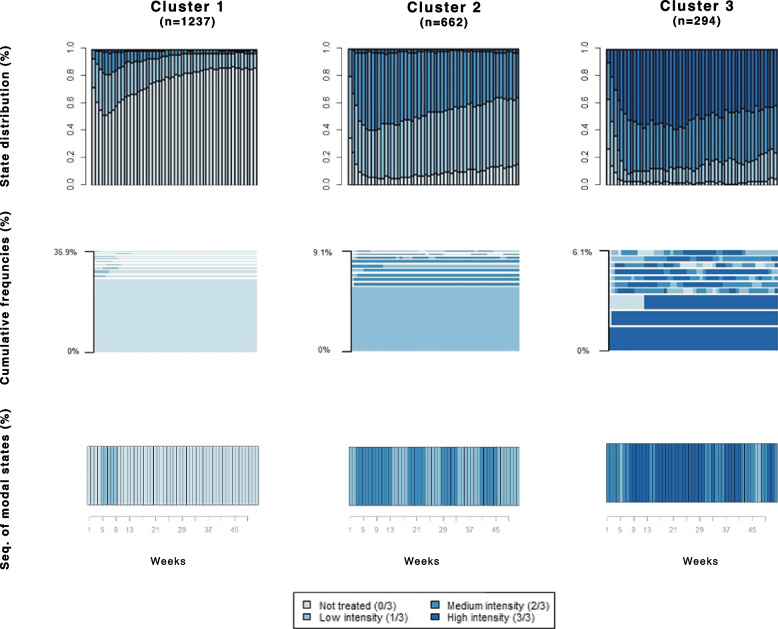
Main plots for the resulted clusters. State distribution plots (first row), frequency plots (second row), and Sequence of modal states plots (third row)

The state distribution plots, the frequency plot, and the sequence made of the modal state at each position, i.e., the typical trajectory, for each cluster (Fig. 5) show the clear differences between patterns followed by the patients of different groups.

### Outcome measure

The results of the cluster analysis were then embraced to predict the time of relapse occurrences, such as emergency hospital admissions reporting a diagnosis of schizophrenia spectrum disorder or an admission in a psychiatric ward. They were labeled outcome episodes and considered measurable surrogates of relapse [41, 42], although the question is still open and debated. The results of the fitted Cox model are reported in Table 6. Compared to patients with a very low variety of treatment (cluster 1), those with medium-low and medium-high variety of treatments showed an increment in the adjusted risk of hospitalization of 80% (95%CI 43%-126% ) and 62% (95%CI 21%-118%), respectively. Being the longitudinal entropy and the turbulence widely explained already by clusters, the probability of being hospitalized seems to be not influenced by them (see the adjusted model). On the contrary, in the first column where the univariable HRs are reported, we see both the clusters and the indices being significantly associated with the outcome.

**Table 6:** Univariable and adjusted hazard ratio (HR), and 95% confidence intervals (CI), of first hospitalization

	Univariable HR (95%CI)	Adjusted HR (95%CI)
Cluster 2^a^	1.81 (1.47 - 2.23)	1.80 (1.43 - 2.26)
Cluster 3^a^	1.93 (1.49 - 2.51)	1.62 (1.21 - 2.18)
High Entropy ^b^	1.36 (1.13 - 1.64)	1.07 (0.83 - 1.38)
High Turbulence ^b^	1.37 (1.13 - 1.66)	0.93 (0.70 - 1.21)

^a^Cluster 1 is the reference; ^b^ Low entropy and low turbulence i.e below the mean, are the references

## Discussion

The aim of the present study was to provide a methodological pipeline based on state sequences for longitudinal care pathway analysis and profiling. This method allows the entire care pathway of each patient to be described as a statistical object and provides both visual and numerical tools for assessment and association with the main endpoint of interest. In addition, SSA makes no assumptions about the data generation mechanism, allowing for a more accurate description and evaluation of events.

In healthcare research based on real-world data, it is common for predictive models to use fixed baseline measures of health care [2, 3]. However, these indices, such as adherence to drug therapy or guideline recommendations, omit valuable information. Indeed, they do not consider the entire evolution of each patient’s clinical picture and are therefore not as informative as they could be. For these reasons, SSA is a way to consider all the longitudinal processes that characterize an individual patient as a mathematical object with a single statistical unit, with the aim of summarizing this complex information with some features that can be inserted into standard predictive models and are able to reflect patient dynamics and behavior. This type of approach leads to more realistic and meaningful results than the commonly used baseline measures, as well as the attempt of some authors who have introduced dynamic monitoring of the effects of adherence to medication effect [43].

Our work case can be a valid example of extrapolating useful and easily readable information about the patient’s path. It can be concluded that the method used allows the phenomenon to be quantified longitudinally, leading to a quantitative identification of the remaining support gap for these patients. For example, cluster 1, which is representative of most patients, has a low diversity of treatments. This means that the quality of care in this cluster is likely lacking, even though we are examining the pathway of young people with the first episode of a serious illness who should receive the best treatment. This means the data can be viewed as a real-world clinical practice feedback tool for decision-making processes.

Some limitations have to be considered when interpreting the results of the sequence clustering analyses. Firstly, the study relies on routinely collected healthcare data for administrative purposes. For this reason, the Lombardy database does not include, for example, several clinical information such as blood pressure, body mass index, smoking status, and diet. Therefore, it is possible that a clinical imbalance between the adherence groups could have influenced the results. However, we took measures to address this issue by adjusting our data for various potential confounding factors. Moreover, the nature of the dataset can also introduce exposure misclassification, as the duration of dispensed drugs is calculated based on defined daily dose metrics, and adherence to treatment was determined based on drug prescriptions assuming that the days covered by a prescription correspond to the actual days of drug use [4]. However, this assumption does not hold true for all patients, leading to overestimating adherence levels in our treatment data. On the other hand, one notable aspect of this kind of database is that it utilized a large and unselected population, thanks to the extensive coverage of the Italian healthcare system for nearly all citizens, making selection bias difficult [4]. Additionally, the drug prescription database we employed ensured accurate data as pharmacists and facilities providing outpatient services are legally obligated to provide detailed reports for reimbursement purposes, thereby minimizing the risk of incorrect information [44]. Secondly, the study’s observation period was restricted to one year to ensure a clear presentation of the different sequences within and between clusters. While significant changes in the observed patterns are not expected, further analysis should apply sequence clustering techniques to a longer observation period to examine the long-term effects of different treatment sequences. Thirdly, the extraction of representative sequences aimed to showcase the essential characteristics of the clusters results in excluding the less frequent pathways in the presentation. This means that some of the captured heterogeneity in the patients’ care pathways may be lost after clustering. Fourth, the choice of dissimilarity measures and clustering algorithm is arbitrary and the results of the analysis may vary depending on these choices. There are no results that prove that one method is superior to others. Therefore sensitivity analysis and domain knowledge are required. Finally, although evidence of effective treatment pathways is needed, the major limitation in the area selected for application is that it is difficult to define a true indicator of the effectiveness of the therapeutic pathway. This is due to the difficulty in finding a consistent definition of the adverse outcome that can truly indicate an exacerbation of the mental illness. It is sufficient to consider that the information on comorbidities is obtained only from the hospitalizations of patients, thus identifying only severe adverse events. Indeed, it is necessary to validate the identified clusters, that is, to verify whether intensification of treatments leads to better outcomes and to identify the appropriate outcomes for this evaluation. The identified clusters indeed seem to group patients with rather similar courses, but in a counterintuitive way, we found that the patients who received more treatment had a higher risk of hospitalization. Given the impossibility of adjusting Cox regression for relevant data such as the severity of the mental illness at entry into the cohort and lifestyle factors (among others), it is likely that a cohort approach could result in estimates that are influenced by confounding. In other words, as pointed out by Corrao et al [38], because patients who have more severe diagnosis of schizophrenia spectrum disorder at baseline are more likely to receive timely and continuing treatment but are also more likely to relapse, this simple design may lead to a paradoxical positive association between intensity of mental healthcare and the occurrence of outcomes. However, the SSA method can be readily applied in traditional cohort and case-control studies, as well as in self-matched studies such as case-crossover or self-controlled case series [45–48]. From a mental health perspective, this work has helped to highlight several important concepts: (i) data-driven techniques can potentially support empirical identification of effective treatment sequences by extracting them from data regularly collected in health care settings, (ii) SSA can be a tool to provide insights into the interval and timing of treatment patterns used in practice and the effectiveness of different treatment sequences. This knowledge could be used to monitor, evaluate, and possibly (re)design optimized treatment pathways. However, it is necessary to identify appropriate indicators for which qualitatively acceptable data are available in order to implement them.

This research represents an important first step in the evaluation of care pathways and paves the way for many further developments. These include model-based approaches such as latent-class analysis [49], which allows direct modeling of sequences (thought of as objects) [50], and the finite-mixture exponential-distance model [51], which allows covariates to influence soft cluster membership probabilities rather than being used for a posteriori profiling of clusters. This second approach is of great interest if the main goal is to better understand whether and to what extent the typical sequence patterns that characterize each cluster are influenced by specific covariates. In addition, a design study must be found to adequately validate the clusters. Furthermore, SSA is a flexible method that can be adapted to different epidemiological contexts thanks to the numerous options for defining states, temporal granularity, and metrics used for spacing. In addition, an interesting development in sequence analysis is the so-called multichannel sequence analysis; this method is an extension of the conventional SSA presented in this article, and it allows to describe individual trajectories on several dimensions simultaneously [52].

## Conclusions

In conclusion, the analysis of state sequences is useful and effective in healthcare research because it offers a valuable gain in pattern discovery. Furthermore, including covariates could establish why specific sequence patterns exist and allow the researcher to capture the potential predictive effect that a specific treatment pathway has on future outcomes. Therefore, SSA could be an effective tool supporting providers in assessing the quality and effectiveness of the diagnostic-therapeutic pathways provided to these patients and monitoring them over time.

## Data Availability

The data that support the findings of this study are available from the Lombardy Region, but restrictions apply to the availability of these data, which were used under license for the current study, and so are not publicly available. Data are however available from the authors upon reasonable request and with permission of Lombardy Region.

## Abbreviations

SSA: State Sequences Analysis
HCU: Healthcare utilization
OM: Optimal Matching
LCS: Longest Common Subsequence
NHS: National Health Service
DMHs: Departments of Mental Health
OTP: Optimal Treatment Pathway
MCS: Multisource Comorbidity Score
